# Erenumab and galcanezumab in chronic migraine prevention: effects after treatment termination

**DOI:** 10.1186/s10194-019-1018-8

**Published:** 2019-06-03

**Authors:** Bianca Raffaelli, Valeria Mussetto, Heike Israel, Lars Neeb, Uwe Reuter

**Affiliations:** 1 Department of Neurology, Charité Universitätsmedizin Berlin, Charitéplatz 1, 10117 Berlin, Germany; 2 Berlin Institute of Health (BIH), Berlin, Germany

**Keywords:** Calcitonin gene-related peptide, Chronic migraine, Erenumab, Galcazenumab, Migraine, Prevention

## Abstract

**Background:**

Monoclonal antibodies (mAbs) targeting the CGRP pathway are safe and efficacious therapies for the prevention of migraine. In this study we assessed the effects of discontinuation of preventive erenumab and galcanezumab treatment in patients with chronic migraine.

**Methods:**

This retrospective pooled analysis included completers of the open-label extension study phase for the preventive treatment of chronic migraine with galcanezumab (NCT02614261; 9 months) and erenumab (NCT02174861; 12 months) in a single headache center. We compare migraine data until week 12 after open-label treatment completion, when patients did not have any pharmacological preventive medication, to study baseline values of the double-blind trial period, and to the last 4 weeks of the open-label extension. The assessment included changes in monthly migraine days, headache hours, days with severe headache and acute headache medication use.

**Results:**

Data from 16 patients after galcanezumab (*n* = 9) and erenumab (*n* = 7) open-label treatment completion were analyzed. The mean number of monthly migraine days was 18.38 ± 3.74 at baseline, and 12.19 ± 4.53 in the last 4 weeks of the open-label extension (*p* < 0.001). Monthly migraine days remained significantly reduced compared to baseline during the entire 12-week observation period after open-label termination (*p* = 0.002), with a reduction of 5.38 ± 4.92 in weeks 1–4 (*p* = 0.001), 4.75 ± 4.15 in weeks 5–8 (*p* = 0.001), and 3.93 ± 5.45 in weeks 9–12 (*p* = 0.014). There was no significant difference in monthly migraine days between the 12 weeks after open-label termination and the last 4 weeks of the open-label phase (*p* = 0.228). All other analyses revealed numerical improvement through week 12 in comparison to baseline.

**Conclusions:**

In this small, self-selected cohort, the results indicate a therapeutic effect of monoclonal antibodies targeting the CRGP pathway in chronic migraine prevention after treatment termination up to 12 weeks.

## Background

Monoclonal antibodies (mAbs) targeting the CGRP pathway are safe and efficacious therapies for the prevention of migraine [1, 2]. Erenumab blocks the CGRP receptor, while galcanezumab, fremanezumab, and eptinezumab bind to the CGRP peptide [3].

Erenumab (NCT02174861) and galcanezumab (REGAIN, NCT02614261) showed a significant reduction of monthly migraine days in chronic migraine during the 3-month double-blind placebo-controlled treatment phase [1, 2]. Both trials included an open-label extension of 9 months in the REGAIN study and 12 months in the erenumab trial [1, 4].

General guidelines for migraine prophylaxis suggest to pause medication after 6–12 months to reevaluate treatment indication [5, 6]. In clinical practice, patients often suffer from a rebound phenomenon, i.e. a renewed increase of migraine frequency after the termination of prophylactic therapy [5, 7].

We assessed the course of chronic migraine following the termination of preventive open-label therapy with erenumab and galcanezumab, when patients were without any preventative medication.

## Methods

We analyzed pooled data from patients in our headache center who completed the galcanezumab and erenumab studies for the prevention of chronic migraine. Trial design for galcanezumab (NCT02614261) included a 3-month double-blind treatment phase followed by a 9-month open-label extension. Patients received monthly s.c. injections of 120 mg or 240 mg galcanezumab during the open-label extension at the discretion of the investigator [1].

Erenumab was studied in a randomized, double-blind, placebo-controlled trial (NCT02174861). Completers of the 12-week double-blind phase could switch to a 52-week open-label extension and received initially a monthly dose of 70 mg erenumab s.c. and, after a protocol amendment, 140 mg [2, 4]. Study details are published elsewhere [1, 2, 4].

This analysis included data from patients who recorded routine headache data for at least 12 weeks after termination of the open-label study phase, and also did not receive any migraine prophylactic medication during this period. We collected data for galcanezumab using the patients’ electronic trial diary during baseline (4 weeks before randomization), the last 4 weeks of the open-label extension, and up to week 12 after open-label termination (i.e. weeks 5–16 after last study drug injection). For erenumab, we analyzed electronic records for the 4-week baseline period prior to randomization and the last 4 weeks of the open-label extension, whereas data from week 1 to week 12 after open-label termination (i.e. weeks 5–16 after last study drug injection) were collected from standardized paper headache diaries. While the electronic diary allowed the precise documentation of headache hours on a minute base, the used paper headache diary collects headache duration in 4-h blocks, data for autonomic symptoms, pain intensity and character, and acute medication use.

We classified acute headache as migraine if the patient had at least two of the following symptoms during the attack: unilateral location, pulsating character, photophobia, phonophobia, nausea/vomiting, aggravation by daily activities, aura and/or improvement after triptan intake.

Statistical analyses were performed using IBM SPSS Statistics, version 24. We compared headache characteristics over time using repeated measures ANOVA with post-hoc paired two-tailed t test, with a *p* value < 0.05 considered statistically significant.

## Results

16/19 patients (84.2%) completed open-label treatment with galcanezumab (9/9) and erenumab (7/10) and presented 3 months later with complete headache diaries. These were mostly female (87.5%) with a mean age of 44.8 (±8.9) years.

**Fig. 1:**
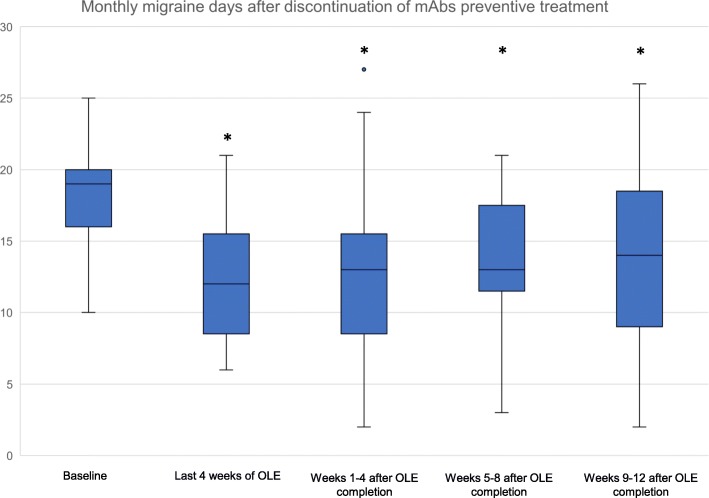
Monthly migraine days during baseline, the last 4 weeks of open-label extension (OLE) and the observation period of 12 weeks-following OLE completion (pooled data). * = significant vs. baseline

At baseline, patients recorded 18.38 (±3.74) monthly migraine days. During the entire observation period monthly migraine days were significantly lower than during baseline (*p* = 0.002) with 12.19 (±4.53) monthly migraine days in the last 4 weeks of the open-label study phases (− 6.19 ± 3.43; *p* < 0.001 vs. baseline), 13.00 (±6.61) in weeks 1–4 after open-label study termination (− 5.38 ± 4.92; *p* = 0.001), 13.81 (±5.96) in weeks 5–8 (− 4.75 ± 4.15; *p* = 0.001), and 14.20 (±6.88) in weeks 9–12 (− 3.93 ± 5.45; *p* = 0.014) (Fig. 1). The analysis of the follow-up period (after completion of the open-label extension) revealed no significant increase in monthly migraine days compared to the last 4 weeks of the open-label phase (*p* = 0.228) as illustrated in Table 1.

**Table 1:** Headache characteristics during baseline, the last 4 weeks of open-label extension (OLE) and the observation period of 12 weeks following OLE termination (pooled data)

	Baseline (before randomization)	Last 4 weeks of OLE	Week 1–4 after OLE completion	Week 5–8 after OLE completion	Week 9–12 after OLE completion
Monthly migraine days					
	18.38 ± 3.74	12.19^a^ ± 4.53	13.00^a^ ± 6.61	13.81^a^ ± 5.96	14.20^a^ ± 6.88
*p* value vs. baseline		< 0.001	0.001	0.001	0.014
*p* value vs. last 4 weeks of OLE			0.539	0.154	0.299
Change in monthly migraine days (vs. baseline)					
	n.a.	−6.19^a^ ± 3.43	−5.38^a^ ± 4.92	−4.75^a^ ± 4.15	−3.93 ^a^ ± 5.46
*p* value		< 0.001	0.001	0.001	0.014
Change in monthly migraine days (vs. last 4 weeks of OLE)					
	n.a.	n.a.	+ 0.81 ± 5.17	+ 1.62 ± 4.33	+ 1.87 ± 6.70
*p* value			0.539	0.154	0.299
Headache hours					
	140.56 ± 80.49	90.75^a^ ± 57.08	91.80^a^ ± 66.58	108.13 ± 69.90	113.60 ± 82.94
*p* value vs. baseline		0.004	0.017	0.067	0.147
*p* value vs. last 4 weeks of OLE			0.917	0.098	0.191
Days with severe headache					
	5.38 ± 4.06	1.69^a^ ± 1.70	2.21^a^ ± 3.26	3.50 ± 4.45	3.86^a^ ± 3.37
*p* value vs. baseline		0.003	0.003	0.069	0.048
*p* value vs. last 4 weeks of OLE			0.683	0.165	0.039
Days with acute headache medication use					
	12.75 ± 5.16	8.94^a^ ± 5.60	10.38^a^ ± 7.50	10.69 ± 7.28	10.67 ± 7.87
*p* value vs. baseline		0.001	0.032	0.085	0.118
*p* value vs. last 4 weeks of OLE			0.179	0.140	0.305
Days with triptan use					
	10.25 ± 7.38	6.06^a^ ± 6.62	6.88^a^ ± 8.28	8.06 ± 8.52	8.00 ± 8.21
*p* value vs. baseline		0.001	0.013	0.115	0.126
*p* value vs. last 4 weeks of OLE			0.327	0.032	0.114

*n.a.* Not applicable

^a^significant vs. baseline

Ten out of 16 patients had less migraine days at all time points compared to baseline. Four chronic migraine patients recorded ≤15 monthly migraine days during the entire observation period, while only two patients had an increase of ≥2 migraine days in weeks 9–12 vs. baseline.

Headache hours, days with severe headache intensity (defined as pain ≥7/10 on a numeric analog scale), days with acute headache medication use and days with triptan use showed a trend to increase over time after trial termination but were still numerically lower than baseline at all examined time points (Table 1).

Data analysis revealed the following two parameters significantly increased compared to the last 4 open-label weeks: number of days with severe headache intensity in weeks 9–12 after termination of the open-label phase (*p* = 0.039) and days with triptan use in weeks 5–8 (*p* = 0.032) (Table 1).

## Discussion

Termination of preventive treatment with galcanezumab and erenumab in patients with chronic migraine did not lead to headache return to baseline levels within a period of 12 weeks. Monthly migraine days remained significantly lower during the entire observation period compared to baseline with a small increase over time. When compared to the last 4 weeks of the open-label phase, our analysis did not indicate any significant worsening of monthly migraine days in the follow-up period. Headache hours, days with severe headache, days with acute medication use and days with triptan use were numerically reduced at all time points compared to baseline, although not statistically significant after week 4.

Persistent benefits up to 16 weeks after the last mAbs injection suggest an interference of mAbs in pathophysiological mechanisms which go beyond the actual treatment period. Both galcanezumab and erenumab have a half-life of ~ 28 days [8, 9]. Plasma concentration after an injection is thus supposed to decrease to 50% within 4 weeks, 25% within eight and 12.5% within 12. Notably, 21 mg of erenumab was without efficacy (vs. placebo) in the phase II episodic migraine study [10]. 50 mg of galcanezumab reduced the free CGRP concentration by only 39% (vs. 76% for 240 mg) [11]. In the phase II trial for galcanezumab in the prevention of episodic migraine, the 50-mg dose did not meet the primary endpoint, defined as reduction of monthly migraine days in the third treatment month vs. baseline [12]. Even though a possible therapeutic effect with low plasma concentration of mAbs cannot be fully excluded, the long lasting beneficial effects are probably not related to direct action of these drugs.

Long-time management of migraine prophylaxis is based on recommendations. The PROMPT study examined migraine relapse after discontinuation of topiramate [5]. Patients with 6 month topiramate treatment reported prolonged benefits after therapy discontinuation and switching to placebo [5]. However, the number of migraine days increased over time, beginning within the first 4 weeks on placebo and was significantly higher than in patients who continued topiramate prophylactic treatment [5]. This study involved patients with episodic migraine, and no subject was without any medication [5]. Considering the short elimination half-time of topiramate (21 h) [13], its beneficial effects over time suggest a modulation of pathophysiological mechanisms of migraine which leads to protracted benefits after treatment termination. A similar phenomenon may apply to CGRP mAbs.

Andreou et al. analyzed the effects of BoNTA discontinuation in clinical practice [7]. Sixty-eight of 200 patients reverted to an episodic migraine pattern after two treatment cycles and were willing to stop treatment. Only 9.3% of these patients remained in an episodic pattern for 12 months after discontinuation, whereas 90.7% worsened and resumed preventive treatment [7].

First real-life data on the use of mAbs as migraine preventive medication revealed a relevant decrease in monthly migraine days after only 1 month of treatment: In an Italian cohort, 65 patients with chronic migraine treated with erenumab reported a reduction of 12.2 monthly migraine days after 4 weeks, along with a decrease in medication use, pain intensity, and disability [14]. While the good efficacy and rapid onset of action of mAbs are meanwhile well-established, there is a lack of scientific data when mAbs treatment should be terminated and concerning long-term effects after treatment termination. This is the first description of headache patterns after discontinuation of treatment with a CGRP ligand and a CGRP receptor antibody, when patients were without any preventative medication or placebo. While previous studies on discontinuation of preventative treatment in chronic migraine focused only on migraine days, we considered several other parameters and showed a numerical improvement in all other measures for the entire observation period.

The limitations of our analysis are the small sample size from a single specialized headache center and the pooling of data from two different mAbs trials. These results should be confirmed in a larger analysis. Notably, the comparison between erenumab and galcanezumab was not within the scope of this study, which allowed pooling of data.

## Conclusions

In conclusion, our results suggest continuous efficacy of mAbs against CGRP/CGRP receptor in the prevention of chronic migraine up to 12 weeks after treatment discontinuation. Monitoring the headache pattern by headache calendars is an essential tool to determine the most suitable long-term therapy strategy for each patient. Data on lager populations in clinical practice over extensive time periods are needed to confirm our results and develop an evidence-based guideline on management of CGRP antibodies over time.

## Data Availability

The datasets analyzed during the current study are available from the corresponding author on reasonable request.

## Abbreviations

BoNTA: Onabotulinumtoxin A
CGRP: Calcitonin gene-related peptide
mAb: Monoclonal antibody
OLE: Open-label extension
s.c.: Subcutaneous

## References

[1] Detke HC, Goadsby PJ, Wang S (2018). Galcanezumab in chronic migraine: the randomized, double-blind, placebo-controlled REGAIN study. Neurology.

[2] Tepper S, Ashina M, Reuter U (2017). Safety and efficacy of erenumab for preventive treatment of chronic migraine: a randomised, double-blind, placebo-controlled phase 2 trial. Lancet Neurol.

[3] Pellesi L, Guerzoni S, Pini LA (2017). Spotlight on anti-CGRP monoclonal antibodies in migraine: the clinical evidence to date. Clin Pharmacol Drug Dev.

[4] Amgen. Clinical Trial Summary: 20130255. Available at: http://www.amgentrials.com/amgen/trialsummary.aspx?studyid=20130255 Accessed 23 Mar 2019

[5] Diener HC, Agosti R, Allais G (2007). Cessation versus continuation of 6-month migraine preventive therapy with topiramate (PROMPT): a randomised, double-blind, placebo-controlled trial. Lancet Neurol.

[6] Sacco S, Bendtsen L, Ashina M (2019). European headache federation guideline on the use of monoclonal antibodies acting on the calcitonin gene related peptide or its receptor for migraine prevention. J Headache Pain.

[7] Andreou AP, Trimboli M, Al-Kaisy A (2018). Prospective real-world analysis of OnabotulinumtoxinA in chronic migraine post-National Institute for health and care excellence UK technology appraisal. Eur J Neurol.

[8] Dodick DW, Goadsby PJ, Spierings ELH (2014). Safety and efficacy of LY2951742, a monoclonal antibody to calcitonin gene-related peptide, for the prevention of migraine: a phase 2, randomised, double-blind, placebo-controlled study. Lancet Neurol.

[9] European Medicines Agency (EMA). Erenumab – product information: summary of product characteristics. Available at: https://www.ema.europa.eu/documents/product-information/aimovig-epar-product-information_en.pdf Accessed 23 Mar 2019

[10] Sun H, Dodick DW, Silberstein S (2016). Safety and efficacy of AMG 334 for prevention of episodic migraine: a randomised, double-blind, placebo-controlled, phase 2 trial. Lancet Neurol.

[11] Kielbasa W, Helton DL (2019) A new era for migraine: pharmacokinetic and pharmacodynamic insights into monoclonal antibodies with a focus on galcanezumab, an anti-CGRP antibody. Cephalalgia:333102419840780. 10.1177/0333102419840780 10.1177/0333102419840780 PMC6710614 30917684

[12] Skljarevski V, Oakes TM, Zhang Q (2018). Effect of different doses of Galcanezumab vs placebo for episodic migraine prevention - a randomized clinical trial. JAMA Neurol.

[13] Doose DR, Walker SA, Gisclon LG, Nayak RK (1996). Single-dose pharmacokinetics and effect of food on the bioavailability of topiramate, a novel antiepileptic drug. J Clin Pharmacol.

[14] Barbanti Piero, Aurilia Cinzia, Egeo Gabriella, Fofi Luisa (2019). Erenumab: from scientific evidence to clinical practice—the first Italian real-life data. Neurological Sciences.

